# Macrophage Subpopulations and the Acute Inflammatory Response of Elderly Human Skeletal Muscle to Physiological Resistance Exercise

**DOI:** 10.3389/fphys.2020.00811

**Published:** 2020-07-24

**Authors:** Simon M. Jensen, Cecilie J. L. Bechshøft, Mette F. Heisterberg, Peter Schjerling, Jesper L. Andersen, Michael Kjaer, Abigail L. Mackey

**Affiliations:** ^1^Institute of Sports Medicine Copenhagen, Department of Orthopedic Surgery M, Bispebjerg Hospital, Copenhagen, Denmark; ^2^Center for Healthy Aging, Faculty of Health and Medical Sciences, University of Copenhagen, Copenhagen, Denmark; ^3^Xlab, Department of Biomedical Sciences, Faculty of Health and Medical Sciences, University of Copenhagen, Copenhagen, Denmark

**Keywords:** macrophages, human skeletal muscle, resistance exercise, sarcopenia, COL1A1, CD68, CD11b, CD206

## Abstract

The current model for repair of damaged tissue includes immune cells, mediating the progression from a pro-inflammatory to an anti-inflammatory environment. How this process changes with aging in human skeletal muscle under conditions of physiological exercise loading remains unclear. To investigate this, 25 elderly males (mean age 70 ± SD 7 years), as well as 12 young (23 ± 3 years) and 12 elderly (74 ± 3 years) females, performed a unilateral bout of heavy resistance leg extension exercise. Biopsies were collected from the vastus lateralis muscle of the rested (control) leg, and post exercise from the exercised leg at 4.5 h, and on days 1, 4, and 7 for the male participants, or on day 5 for the female participants. Total macrophages (CD68+) as well as pro- (CD11b+) and anti-inflammatory (CD163+, CD206+) subpopulations were identified on sections by immunohistochemistry. Gene expression levels of COL1A1, TNF-a, CD68, myostatin, TCF7L2, IL-1B, IL-1R, IL-10, and Ki67 were determined by real-time RT-PCR. At rest, the muscle tissue from the elderly vs. young females was characterized by higher gene expression levels of CD68, IL-10, lower myostatin mRNA, and trends for a greater number of macrophages, while COL1A1 mRNA post exercise values were greater in the elderly vs young. For the male participants, mRNA levels of the inflammatory cytokines IL-1B, IL-1R were elevated in the early phase following exercise, followed by increases in COL1A1 and Ki67 on days 4 and 7. In general, exercise induced increases in all types of macrophages counted in the elderly, but not in young, individuals. Cells expressing CD68, CD11b, and CD206 simultaneously were the most frequently observed cell type, which raises the possibility that pure pro- and anti-inflammatory macrophages populations do not exist in healthy human skeletal muscle within the spectrum of tissue remodeling induced by physiological exercise designed to induce hypertrophy. Together these data provide insight into the time course of macrophage activity and associated molecular targets in human skeletal muscle in the context of aging and exercise.

## Introduction

Skeletal muscle is a plastic tissue with a significant capacity to regenerate and adapt following severe damage, even in elderly individuals in their 60s (Karlsen et al., 2020). The developing model for repair of damaged tissue includes immune cells, mediating the progression from a pro-inflammatory response to an anti-inflammatory state (Arnold et al., 2007; Tidball and Villalta, 2010; Saclier et al., 2013a). This process is precisely regulated and is crucial for proper regeneration, as evidenced in studies perturbing the infiltration sequence to later identify altered and impaired muscle regeneration (Chazaud, 2014). However, it appears that a certain degree of dysregulation of the inflammatory response exists in elderly individuals (Reidy et al., 2019) which could be a potential factor in the age-related loss of skeletal muscle mass. Furthermore, in patients with peripheral artery disease, low macrophage content of the gastrocnemius muscle was found to be associated with better walking performance (Kosmac et al., 2020) suggestive of potential clinical implications for macrophages in human skeletal muscle.

Following muscle damage, resident macrophages are believed to orchestrate the attraction of monocytes to the site of injury (Brigitte et al., 2010) where the local environment will drive the monocyte toward a macrophage phenotype (Kumar and Jack, 2006; Murray and Wynn, 2012). Macrophage phenotypes are generally classified into two opposing categories: (1) pro-inflammatory macrophages (M1) containing phagocytic properties, enhanced microbicidal capacity and a high secretion of pro-inflammatory cytokines (IL-1B and TNF-a), or (2) anti-inflammatory macrophages (M2) associated with anti-apoptosis, tissue repair and growth factors (Mosser and Edwards, 2008). These characteristics are mostly described from *in vitro* studies and likely represent the extremes in a spectrum of many possible phenotypes (Saclier et al., 2013b). Cytokines expressed by macrophages have also been shown to regulate myogenic precursor cell fate (Arnold et al., 2007; Saclier et al., 2013a; Chazaud, 2014) further supporting a role for macrophages as key players in muscle regeneration, repair and maintenance.

Muscle regeneration, as defined by events following myonecrosis (Grounds, 2014) is, however, an extreme state of tissue remodeling, compared to the remodeling induced by typical physiological exercise regimens performed by individuals throughout the lifespan. Most information about macrophages has been elucidated from very invasive animal studies and *in vitro* studies, and indeed severe eccentric protocols in humans (Jones et al., 1986; Paulsen et al., 2010a, b, 2012a) as reviewed earlier (Paulsen et al., 2012b; Peake et al., 2017). However, there is relatively little knowledge about macrophage infiltration in human skeletal muscle under physiological settings and even fewer studies comparing young and elderly individuals directly. For example, it has been shown that 14 weeks of progressive heavy resistance training induces increases in the number of anti-inflammatory and total number of macrophages in the skeletal muscle of elderly individuals (Walton et al., 2019a). Similarly, in a group of individuals ranging in age from 29 to 68, 12 weeks of endurance cycle training was observed to increase the anti-inflammatory macrophage content of the working muscles, where resident anti-inflammatory muscle macrophages are associated with exercise-mediated increases in skeletal muscle fiber size, suggesting a role in muscle growth (Walton et al., 2019b). Of the few studies comparing young and elderly subjects, recent work has investigated local macrophage content using a muscle damage protocol and found infiltration sequences mimicking the pattern of that seen in animal studies, and also showing differences between young and elderly subjects (Sorensen et al., 2019). This supports the findings from an earlier study where cellular and molecular differences between young and elderly individuals were observed 3 days after a physiological bout of resistance exercise (Przybyla et al., 2006). However, a detailed time-course for inflammatory cell infiltration and the associated molecular response following a physiological bout of resistance exercise remains yet undescribed.

Furthermore, there is a general lack of consensus regarding appropriate markers to distinguish between pro- and anti-inflammatory macrophages on sections of human skeletal muscle. A recent study addressed this methodological issue, proposing an immunohistochemistry protocol, validated by flow cytometry, While CD68 is considered a macrophage pan marker and CD11b has been used as a marker for proinflammatory macrophages (Przybyla et al., 2006; Sorensen et al., 2019) a study by Kosmac et al. (2018) reported that CD68 and CD11b can be used interchangeably as pan markers highlighting the need for continued research and development into methods identifying markers and their specificity to immune cells. This complexity is important to recognize when designing antibody-based protocols. The study by Kosmac also found that the most commonly observed cell type was positive for both CD11b and CD206 and that macrophages in human skeletal muscle in the rested state simultaneously express markers of the so-called classic pro-inflammatory and anti-inflammatory phenotypes (Kosmac et al., 2018) indicating that the current classification may not reflect the true *in vivo* state in healthy human skeletal muscle. Building on these studies, and to address the paucity of knowledge on the time course of the macrophage response in young and elderly healthy individuals, the main purpose of this study was to compare the inflammatory profile of young and elderly skeletal muscle at rest and in response to a single bout of physiological resistance exercise. We hypothesized that, when compared to younger individuals, the skeletal muscle of elderly individuals would be characterized by a greater presence of anti-inflammatory macrophages, in accordance with earlier work (Sorensen et al., 2019) reflected by a corresponding gene expression profile. Furthermore, we hypothesized that the macrophage response to exercise would be dominated by anti-inflammatory macrophages in the elderly, but not young, participants.

## Materials and Methods

### Experimental Design

This study is based on muscle biopsy analyses from two studies, one on 25 elderly men (Heisterberg et al., 2018) and one on 12 young and 12 elderly women (Bechshøft et al., 2019; Soendenbroe et al., 2020). Both studies were approved by The Committees on Health Research Ethics for The Capital Region of Denmark (Ref: H-15017223, H-3-2012-081). All procedures conformed to the Declaration of Helsinki and the subjects gave written informed consent before participation. Generally, all participants in both studies were considered healthy, non-smokers and did not perform any strenuous physical exercise on a regular basis – more information about the participants is available in Table 1. Initially, the men were part of a randomized controlled trial investigating the effect of the blood pressure lowering medication Losartan on the muscle response to exercise in healthy normotensive males, where half of the participants received Losartan and the other half placebo. Given the general lack of any drug effect (Heisterberg et al., 2018) the two groups in the male study were merged in the present study (separate group data are also provided for reference in Supplementary Material).

**TABLE 1:** Participant characteristics.

	Young females		Elderly females		Elderly males	
	*n = 12*		*n = 12*		*n = 25*	
Age (yr)	23 ± 3	[20–28]	74 ± 3	[70–78]	70 ± 7	[64–90]
Height (cm)	168 ± 7	[157–177]	165 ± 3	[159–169]	180 ± 5	[172–189]
Weight (kg)	64 ± 8	[53–75]	68 ± 10	[57–84]	82 ± 10	[67–98]
BMI (kg/m^2^)	23 ± 2	[19–26]	25 ± 4	[20–30]	26 ± 3	[21–31]
Knee extension 1RM (kg)	39 ± 8	[30–50]	24 ± 5	[12–29]	56 ± 14	[23–82]

For both studies, the exercise protocol consisted of one session of unilateral knee extension heavy resistance exercise. The exercised leg was chosen by randomization and the inactive leg served as a control leg. The heavy resistance exercise consisted of a concentric and eccentric exercise bout where load was determined from one repetition max (RM) performed in a leg-extension machine (M52, TechnoGym, Cesena, Italy). The exercise was performed using the same equipment as described in detail (Heisterberg et al., 2018; Bechshøft et al., 2019). Briefly, the men performed 5 sets of 12 concentric repetitions (70% 1RM) followed by 4 sets of 6 eccentric repetitions (110% 1 RM). The exercises were performed with a two-minute break between the sets and five-minute break between the two different types of exercise. The women performed 4 sets of 12 repetitions (70% 1RM) of concentric contractions with a two-minute break between each set. This bout was followed by 4 sets of 4 repetitions (110% 1RM) of eccentric contractions with two-minute breaks between each set. After 5–10-min of resting the women performed the entire protocol a second time.

### Muscle Biopsies

For all participants, muscle biopsies were obtained from the vastus lateralis muscle, under local anesthetic (1% lidocaine), using the percutaneous needle biopsy technique of Bergström, with 5–6 mm needles and manual suction. Pieces of muscle tissue were aligned, embedded in Tissue-Tek, and frozen in isopentane, pre-cooled in liquid nitrogen, and stored at −80°C. The men had six muscle biopsies taken over 17 days, at the following time points: −10 and −3 days before exercise (from the control, non-exercised, leg), and from the exercised leg at +4.5 h, and on days +1, +4, and +7 post exercise. The day −3 sample was excluded from the current study since its purpose was to investigate a potential effect of losartan in the rested state and is therefore irrelevant in the current context. The young and elderly women had muscle biopsies collected from each leg 5 days after exercise.

### RNA-Extraction

Approximately 2–5 mg of muscle tissue (100 cryosections of 10 μm) was homogenized in 1 ml of TriReagent (Molecular Research Center, Cincinnati, OH, United States) containing five stainless-steel balls of 2.3 mm in diameter (BioSpec Products, Bartlesville, OK, United States) by shaking in a FastPrep-24 instrument (MP Biomedicals, Illkirch, France) at speed level 4 for 15 s. Following homogenization, bromo-chloropropane was added to separate the samples into an aqueous phase and an organic phase. Following isolation of the aqueous phase, RNA was precipitated using isopropanol. The RNA pellet was then washed in ethanol and subsequently dissolved in 20 μl of RNAse-free water. Total RNA concentrations and purity were determined by spectroscopy at 260, 280, and 240 nm. Good RNA integrity was ensured by gel electrophoresis.

### Real-Time RT-PCR

Five hundred nanogram of total RNA was converted into cDNA in 20 μl using the OmniScript reverse transcriptase (Qiagen) and 1 μM poly-dT (Invitrogen, Naerum, Denmark) according to the manufacture’s protocol (Qiagen). For each target mRNA, 0.25 μl of cDNA was amplified in a 25-μl SYBR Green polymerase chain reaction (PCR) containing 1 Quantitect SYBR Green Master Mix (Qiagen) and 100 nM of each primer (Table 2). The amplification was monitored in real time using the MX3005P Realtime PCR machine (Stratagene). The CT values were related to a standard curve made with known concentrations of cloned PCR products or DNA oligonucleotides (Ultramer oligos; Integrated DNA Technologies, Leuven, Belgium), with a DNA sequence corresponding to the sequence of the expected PCR product. The specificity of the PCR products was confirmed by melting curve analysis after amplification. RPLP0 mRNA was chosen as internal control. To validate this use, another unrelated “constitutive” mRNA, GAPDH, was measured and normalized with RPLP0 (Heisterberg et al., 2018; Soendenbroe et al., 2020).

**TABLE 2:** PCR primers.

mRNA	NCBI ID	Sense	Anti-sense
RPLP0	NM_053275.3	GGAAACTCTGCATTCTCGCTTCCT	CCAGGACTCGTTTGTACCCGTTG
GAPDH	NM_002046.4	CCTCCTGCACCACCAACTGCTT	GAGGGGCCATCCACAGTCTTCT
COL1A1	NM_000088.3	GGCAACAGCCGCTTCACCTAC	GCGGGAGGACTTGGTGGTTTT
TNFa	NM_000594.3	TTCCCCAGGGACCTCTCTCTAATC	GAGGGTTTGCTACAACATGGGCTAC
CD68	NM_001251.2	CAGCTTTGGATTCATGCAGGACC	CTCTGCCCCAGGGGTGCTTG
Myostatin	NM_005259.2	TGCTGTAACCTTCCCAGGACCA	GCTCATCACAGTCAAGACCAAAATCC
Myogenin	NM_002479.5	CTGCAGTCCAGAGTGGGGCAGT	CTGTAGGGTCAGCCGTGAGCAG
TCF7L2	NM_001146274.1	CGGAAGGAGCGACAGCTTCAT	GTCTCTCCCGGCTGCTTGTCC
IL-1B	NM_000576.2	TGCGTGTTGAAAGATGATAAGCCCA	CAAATCGCTTTTCCATCTTCTTCTTTG
IL-10	NM_000572.3	CGCTGTCATCGATTTCTTCCCTGT	TGGCTTTGTAGATGCCTTTCTCTTGG
IL-1R	NM_000877.4	GGAAGGGATGACTACGTTGGGGA	CCAGCCAGCTGAAGCCTGATGTT
Ki67	NM_002417.4	CGGAAGAGCTGAACAGCAACGA	GCGTCTGGAGCGCAGGGATA

### Immunohistochemistry

Sections (10 μm) of the muscle biopsies were cut at −20°C in a cryostat and placed on glass slides. In the male study immunohistochemical labeling was performed using primary antibodies CD68 and CD163 (Table 3). Briefly, sections were fixed in 5% formaldehyde (Histofix; Histolab, Gothenburg, Sweden) for 10 minutes and refrigerated overnight with 2 primary antibodies in 1% BSA buffer, according to Table 3. The following day 2 secondary antibodies (Table 3) diluted in 1% BSA were added for 45 min. Finally, slides were mounted with Prolong-Gold-Antifade with DAPI (Cat. No. P36931; Molecular Probes/Invitrogen). Between all steps, slides were washed in two changes of 0.05M Tris-buffered Saline (TBS). Slides were stored at room temperature for 24 h, protected from sunlight, and then stored at −20°C until analysis by microscopy. For the samples in the female study the same method was followed but developed further to include three primary antibodies. In order to have three antibodies from three different host species, CD206 was used instead of CD163, as supported elsewhere (Kosmac et al., 2018, 2020) resulting in the following three primary antibodies: CD68, CD11b, and CD206 (Table 3). Other changes to the protocol were that samples were blocked with 5% donkey serum in TBS for 30 min and then refrigerated overnight with three primary antibodies diluted in 1% donkey serum in TBS, according to Table 3. The following day 3 secondary antibodies (Table 3) diluted in 1% donkey serum were added for 60 min, before slides were mounted.

**TABLE 3:** Antibodies.

Males				
Host	Antibody	Company	Cat. no.	Concentration
**Primary antibody**				
Rabbit	CD68	Sigma-Aldrich	HPA048982	1:200
Mouse	CD163	Santa Cruz	SC-2006	1:200

**Host**		**Company**	**Cat. no.**	**Concentration**

**Secondary antibody**				
Goat Anti-rabbit 568		Thermo Scientific	A-11036	1:500
Goat anti-mouse 488		Thermo Scientific	A-1109	1:500

**Females**				

**Host**	**Antibody**	**Company**	**Cat. no.**	**Concentration**

**Primary antibody**				
Mouse	CD68	Dako	M0718	1:500
Rabbit	CD11b	Abcam	AB52478	1:500
Goat	CD206	RD Systems	AF2534	1:1000

**Host**		**Company**	**Cat. no.**	**Concentration**

**Secondary antibody**				
Donkey anti mouse 488		Abcam	Ab-150109	1:200
Donkey anti rabbit 568		Abcam	Ab-175693	1:200
Donkey anti goat 680		Thermo Scientific	A-21084	1:500

### Microscopy

Images from each section were collected using a DP71 Olympus Camera mounted on a BX51 Olympus microscope (x20/0.50NA objective) using the Olympus cellSens software (v.1.14). Image size was 2040 × 1536 pixels, corresponding to 868 × 653 μm. For all sections, the goal was to image areas where transversely aligned fibers filled the entire image, avoiding perimysium where possible since it usually contains higher numbers of macrophages than the endomysium. These areas, along with longitudinally oriented fibers and sectioning artifacts were excluded from the analysis.

The same person conducted image analysis for all participants. Blinded to time, the following sequence of analysis was performed in the male study: number of fibers and area of tissue included in the analysis, cells that were (1) CD68+CD163−, (2) CD68+CD163+, and (3) CD68−CD163+. Each cell was evaluated manually in ImageJ (v.150c4, National Institutes of Health, United States). Cells were only marked positive for a marker if the cell nucleus (DAPI) was centrally located within the area of antibody staining. CD68+ was considered a pan-macrophage marker while CD68+CD163+ cells were considered anti-inflammatory. In Figure 3 CD68+ total is a combination of CD68+CD163− and CD68+CD163+ cells. Pro-inflammatory macrophages were not identified in the male study.

**FIGURE 1:**
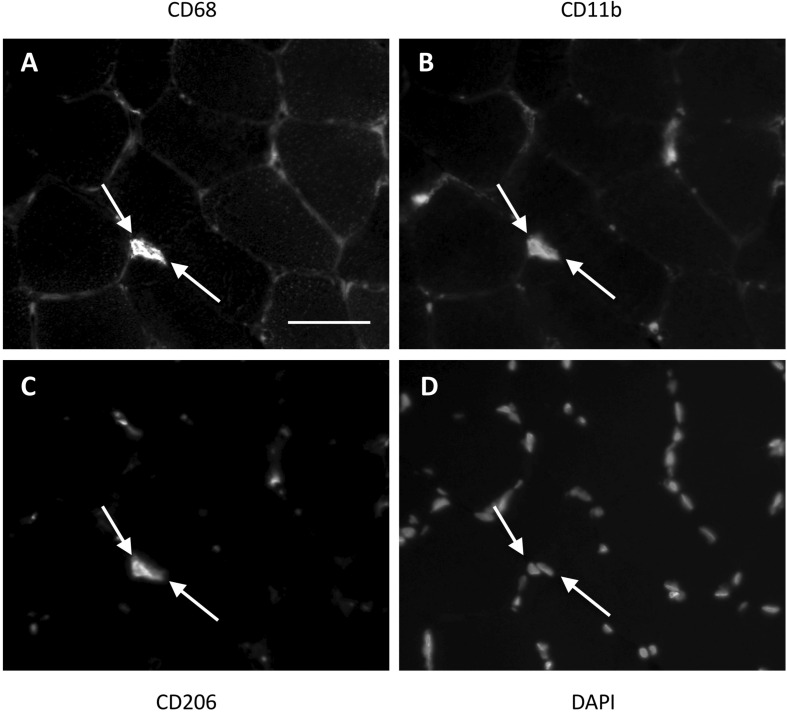
Method for identification of macrophages in the female study. Two triple positive cells (indicated with white arrows) located next to each other in a muscle section. CD68 **(A)** was used as a pan-macrophage marker, CD11b **(B)** as a proinflammatory phenotype marker and CD206 **(C)** as an anti-inflammatory phenotype marker. DAPI **(D)** identifies the cell nucleus. Cells were only marked positive for a marker if the cell nucleus was centrally located within an area of antibody staining. Scale bar indicates a length of 50 μm.

**FIGURE 2:**
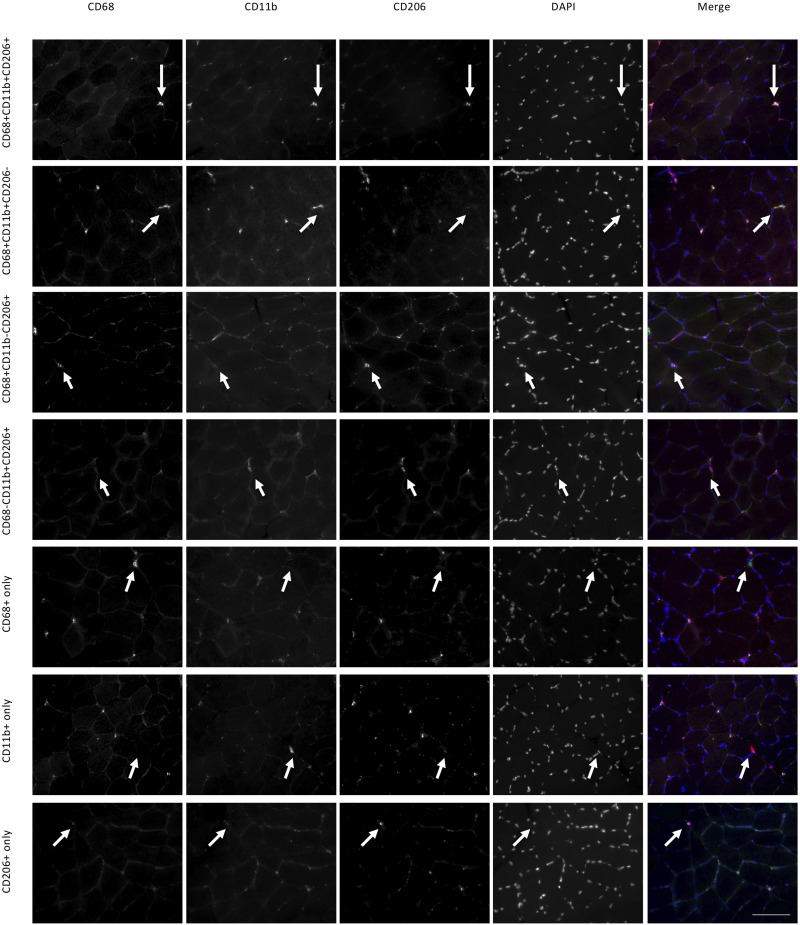
Macrophage histology and identified cell categories in the female study. Each row is a collection of images showing the same area of a muscle section. Using three antibodies (CD68, CD11b, CD206) we identified seven categories of labeled cells, each represented on a different row. For each category white arrows point to the same cell. Scale bar indicates a length of 100 μm.

In the female study, blinded to age and exercise, the following sequence of analysis was performed in addition to fiber count and tissue area: cells that were only positive for either CD68, CD11b or CD206, cells that were CD68+CD11b−CD206+, CD68+CD11b+CD206−, CD68−CD11b+CD206+ (double positive), and cells that were CD68+CD11b+CD206+ (triple positive). Figures 1, 2 show examples of all identified cell categories. CD68 was considered a pan-macrophage marker, while co-localization with CD11b+ or CD206+ was considered pro-inflammatory and anti-inflammatory, respectively. For comparison with other studies, pro (CD68+CD11b+) and anti-inflammatory macrophages (CD68+CD206+) are displayed in addition to CD68+CD11b+CD206+ in Figures 5B,C. Table 4 provides additional information on how cell counts have been classified and combined in the female study. Some macrophage populations were observed in very limited numbers in the male study (CD68−CD163+) and female study (CD68−CD11b+CD206+, CD68−CD11b−CD206+), so these data are not reported.

**TABLE 4:** Cell definition table – female.

Definition	Remark	Subpopulations combined
CD68^+^ total	All cells positive for CD68	CD68^+^ CD11b^–^ CD206^–^ CD68^+^ CD11b^+^ CD206^–^ CD68^+^ CD11b^–^ CD206^+^ CD68^+^ CD11b^+^ CD206^+^
CD68^+^ CD11b^+^	Pro-inflammatory macrophages	CD68^+^ CD11b^+^ CD206^–^ CD68^+^ CD11b^+^ CD206^+^
CD68^+^ CD206^+^	Anti-inflammatory macrophages	CD68^+^ CD11b^–^ CD206^+^ CD68^+^ CD11b^+^ CD206^+^
CD68^+^ CD11b^+^ CD206^+^	Triple positive cells	CD68^+^ CD11b^+^ CD206^+^
CD11b^+^ only	Positive for only CD11b	CD68^–^ CD11b^+^ CD206^–^

All macrophage data are expressed relative to the number of fibers included in each section, or the tissue area.

### Statistics

All figures were prepared in GraphPad Prism (v.7.04, GraphPad Software, Inc., La Jolla, CA, United States) and all statistical analyses were conducted in SigmaPlot (v. 13.0, Systat Software Inc, San Jose, CA, United States), except subject characteristics and gene expression of the female subjects, which was analyzed by *t*-test in Microsoft Excel 2016 (Microsoft Corporation, Redmond, WA, United States), and the Spearman rank correlations, which were performed in GraphPad Prism. *P*-values below 0.05 were considered significant. For both studies, mRNA data were normalized to RPLP0 and log-transformed before statistical analysis. For the male participants, since cell count data were not normally distributed, Friedman Repeated Measures Analysis of Variance on ranks was used, while mRNA data were analyzed using a one-way repeated measures analysis of variance. Dunnett’s *post hoc* test for multiple comparisons was used to compare each time point with baseline (−10 days). Cell count data from the male study are displayed as individual values (cells/fiber) while mRNA data are displayed as geometric mean ± back transformed SEM. The cell count data from the female study were analyzed by Wilcoxon signed rank test to compare data within the same group (trained vs. untrained), while Mann–Whitney Rank sum test was used to compare between groups (young vs. old) in the control leg only. *P*-values were then Bonferroni corrected using a multiplication factor of 3 which is the number of comparisons made. Cell counts are displayed as individual data (cells/fiber). For the female participants, unpaired *t*-tests were performed between young and old for mRNA data while paired *t*-tests were conducted for the mRNA analysis of the exercise response (exercised leg vs. control leg). As for the males, female mRNA data are displayed as geometric mean ± back transformed SEM. Subject characteristics and number of fibers analyzed are presented as means with standard deviation and range.

## Results

### Immunohistochemistry – Male

**FIGURE 3:**
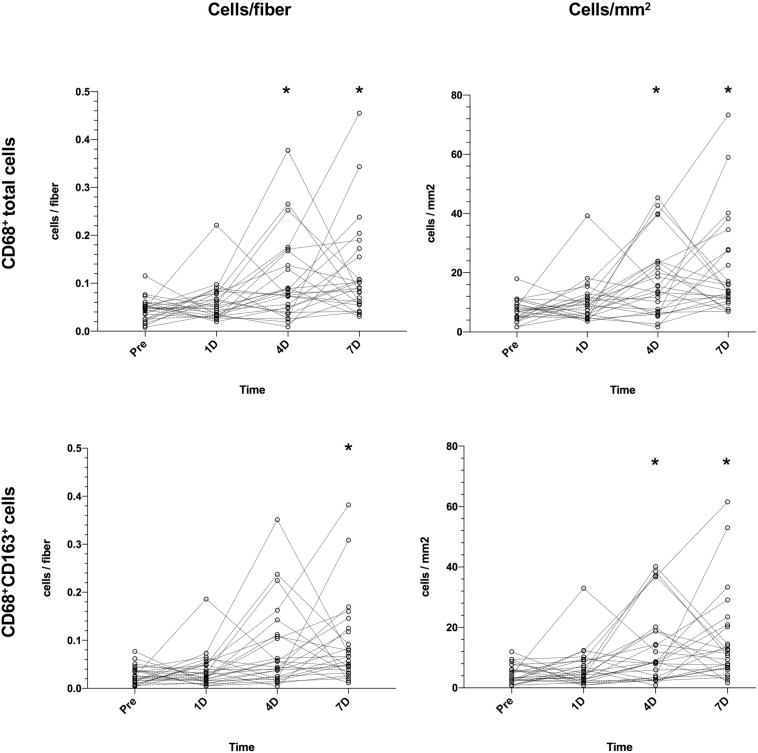
Changes in muscle CD68+ cells **(top)** and CD68+CD163+ cells **(bottom)** in healthy elderly men (*n* = 25) over a 7-day time course following an acute bout of heavy resistance leg extension exercise. Data was analyzed using Friedman repeated measures analysis of variance on ranks and each timepoint was compared with baseline (–10 days) using Dunnett’s *post hoc* test. Individual values are displayed as cells/fiber (left) and cells/mm^2^ (right). *P < 0.05.

Figure 3 illustrates changes in muscle CD68+ cell content/fiber over time after an acute bout of resistance training. PRE values from subjects (0.048 cells/fiber) increased on D4 (0.075 cells/fiber, 56%, *P* = 0.04) and D7 (0.090 cells/fiber, 88%, *P* < 0.001), with 3 participants peaking on D1, 7 on D4, 14 on D7, and 1 participant failing to show any increase. Data for CD68+CD163+ cells show an increase from PRE (0.024 cells/fiber) to D7 (0.066 cells/fiber, 175%, *P* = 0.002). Generally, the same pattern were seen for data expressed as cells/mm^2^. On average, the number of fibers included in the macrophage analysis was 212 ± 58 [98–403].

### mRNA – Male

**FIGURE 4:**
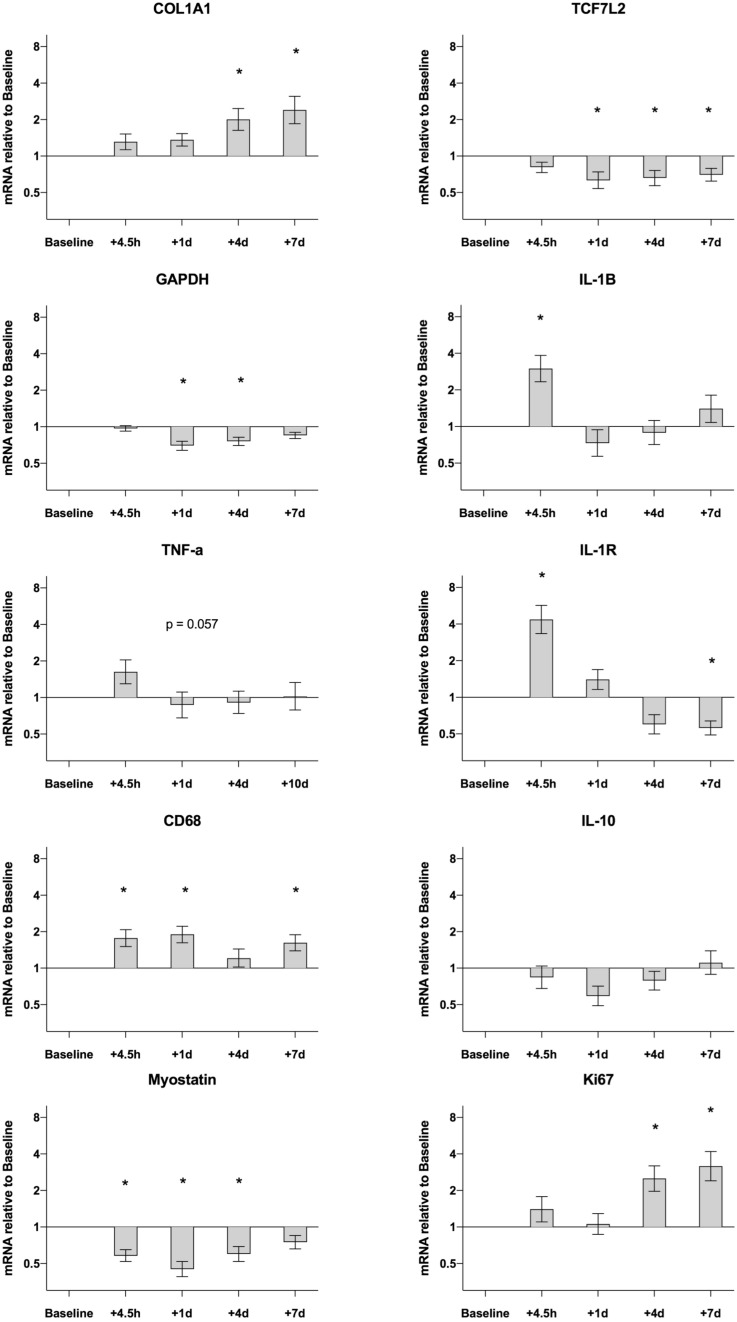
Gene expression in muscle biopsies of healthy elderly men (*n* = 25) following an acute bout of heavy resistance leg extension exercise. mRNA data were normalized to RPLP0 and are shown as geometric mean ± back transformed SEM, relative to baseline (–10 days). Data were analyzed by one-way repeated measure analysis of variance using Dunnett’s *post hoc* test. *P < 0.05. Data are Losartan and Placebo groups merged as shown in Supplementary Figure S2. Separate group data for COL1A1, Myostatin, GAPDH, CD68 and TCF7L2 have been published previously (Heisterberg et al., 2018).

Data are presented in Figure 4. Most of the measured genes demonstrated changes over the 7-day time-course. Compared to pre values gene expression for CD68 was upregulated at 4.5H (1.8-fold, *P* = 0.001), D1 (1.9-fold, *P* < 0.001) and D7 (1.6-fold, *P* = 0.008). Other targets related to the inflammatory response showed an acute increase peaking at 4.5H, then returning to, or dropping below, baseline values. This was evident for IL-1B (4.5H, 3.0-fold, *P* < 0.001) and IL-1R (4.5H, 4.4-fold, *P* < 0.001). No changes were detected for TNF-a and IL-10 over the entire time course. Other targets displayed a somewhat delayed response that could not be detected until D4 and D7. This was the case for COL1A1 (D4, 2.0-fold, *P* = 0.006; D7, 2.4-fold, *P* < 0.001) and Ki67 (D4, 2.5-fold, *P* = 0.007; D7, 3.2-fold, *P* < 0.001). Gene expression for Myostatin was down regulated following the exercise session both at 4.5H (0.6-fold, *P* = 0.002), D1 (0.5-fold, *P* < 0.001) and D4 (0.6-fold, *P* = 0.005). Other targets that were down regulated were GAPDH on D1 (0.7-fold, *P* < 0.001) and D4 (0.8-fold, *P* < 0.001) and TCF7L2 on D1 (0.6-fold, *P* = 0.007) D4 (0.7-fold, *P* = 0.018) and D7 (0.7-fold, *P* = 0.047).

### Immunohistochemistry – Female

**FIGURE 5:**
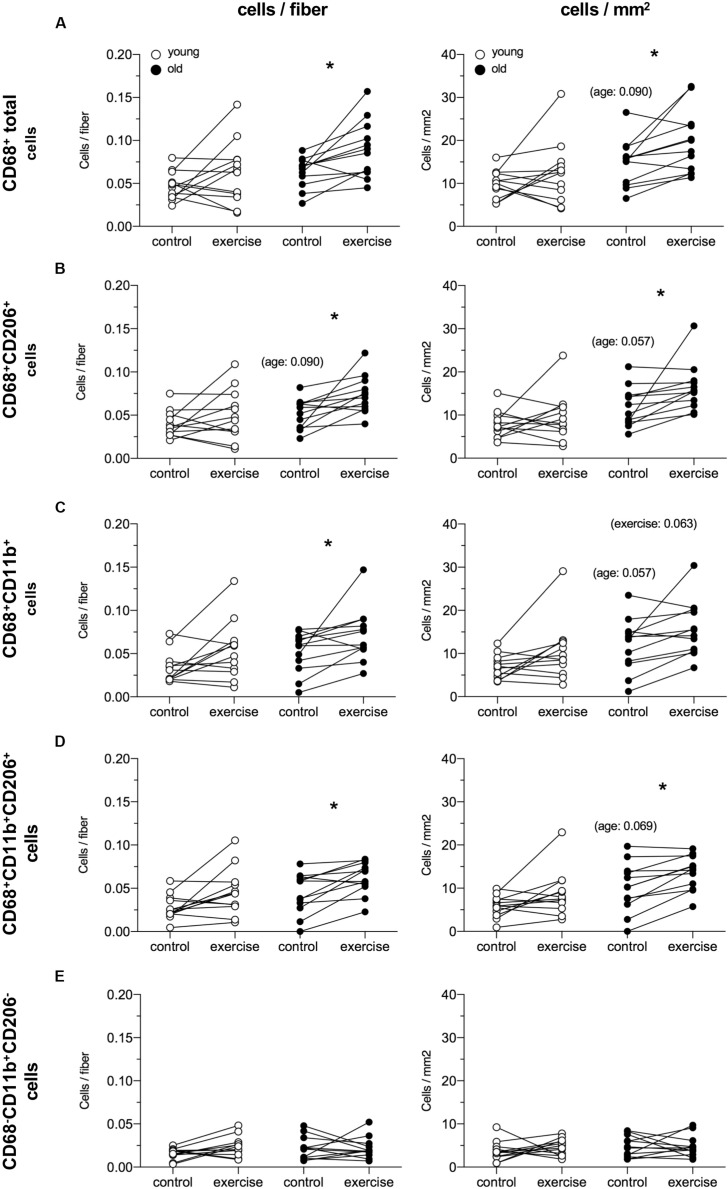
Local macrophage content in healthy young (*n* = 12) and elderly (*n* = 12) women from control and exercised leg 5 days following an acute bout of unilateral resistance exercise. Data are presented as cells/fiber (left) and cells/mm^2^ (right) for CD68+ total cells **(A)**, CD68+CD206+ cells **(B)**, CD68+CD11b+ **(C)**, CD68+CD11b+CD206+ cells **(D)** and CD68-CD11b+CD206- cells **(E)**. Individual data points are displayed. Data were analyzed using Mann–Whitney (young vs. old) and Wilcoxon signed rank test (trained vs. untrained), and *p*-values were Bonferroni corrected (x3). *P < 0.05 vs. control leg. The inserted *p*-values indicate trends.

Data from the female macrophage analysis are shown in Figure 5. The exercised leg in the old group showed an greater number of CD68+ total cells compared to the control leg 5 days after a heavy resistance exercise bout (0.068 vs. 0.088, 29%, *P* = 0.021). When comparing the exercise and control leg in the old group, differences were observed for CD68+CD206+ cells (0.055 vs. 0.072 cells/fiber, 31%, *P* = 0.009), CD68+CD11b+ cells (0.059 vs. 0.068 cells/fiber, 15%, *P* = 0.048) and CD68+CD11b+CD206+ cells (0.048 vs. 0.064 cells/fiber, 33%, *P* = 0.021). No significant differences were observed in the young group. For all cell types, a similar pattern was seen for data expressed as cells/mm^2^, except for CD68+CD11b+ cells where only a trend for a change was exercise was seen in the elderly individuals. Furthermore, trends were detected for greater numbers of all macrophage types, except for CD68−CD11b+CD206−, when expressed per mm^2^ tissue, in the elderly vs. young control leg. The average number of fibers included in the macrophage analysis was 294 ± 74 [189–422] and 294 ± 88 [146–446] for the young and old group, respectively.

### mRNA – Female

**FIGURE 6:**
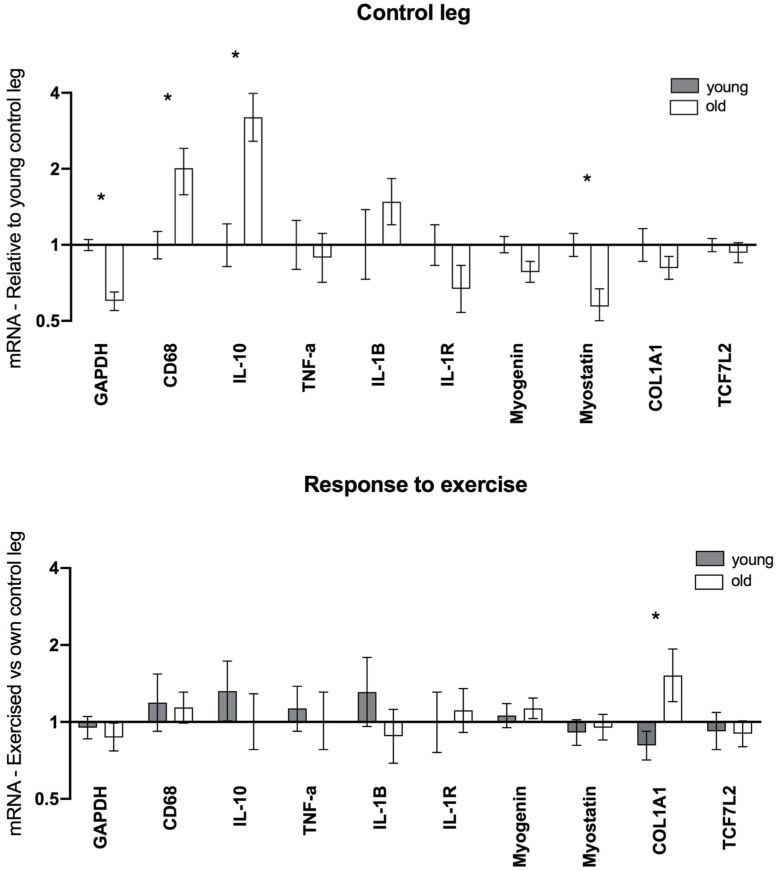
Gene expression in muscle biopsies of healthy young (*n* = 12) and elderly (*n* = 12) women 5 days after a single bout of unilateral resistance exercise. mRNA data were normalized to RPLP0 and shown as geometric mean ± back transformed SEM, relative to young control leg (Control leg) and own control leg (Response to exercise). *P < 0.05 vs. young. GAPDH data have been published previously (Soendenbroe et al., 2020).

Data from the female gene expression analysis are displayed in Figure 6. Elevated levels of CD68 (2.0-fold, *P* = 0.005) and IL-10 (3.2-fold, *P* < 0.001) were found in the old group compared to the young. Additionally, Myostatin (0.6-fold, *P* = 0.007) and GAPDH (0.6-fold, *P* < 0.001) levels were significantly lower in the old group, while no significant differences were observed for other targets. Post exercise, greater levels of COL1A1 were observed in the elderly vs. young individuals. Aside from this no significant differences were observed for any of the analyzed targets.

### Macrophages Correlations With BMI and 1RM

**FIGURE 7:**
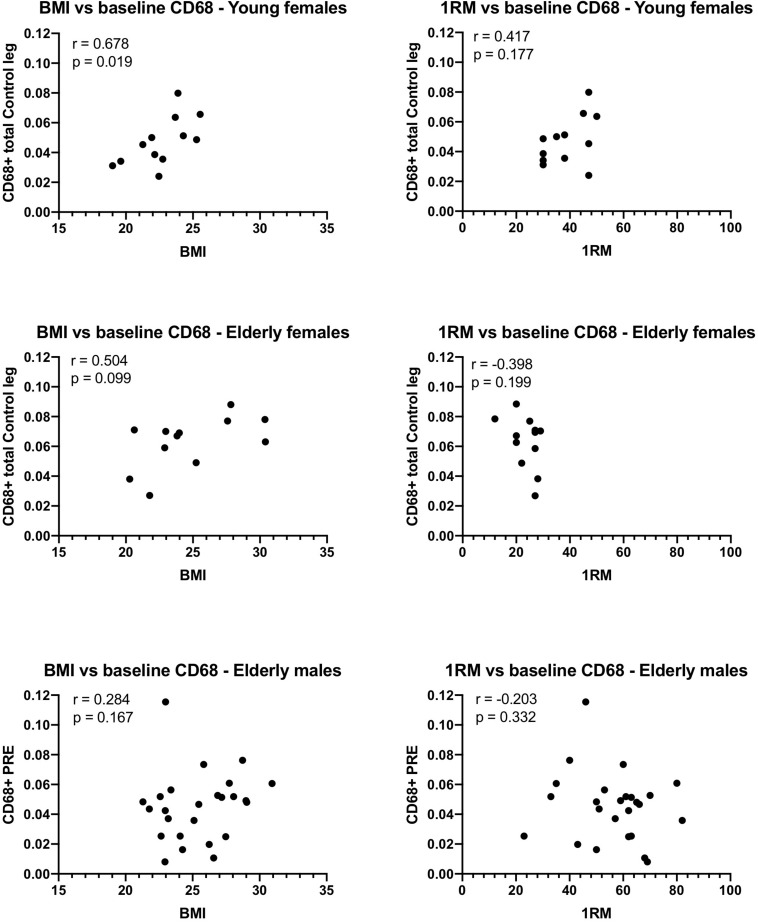
Relationship between body mass index (BMI), or 1-repition maximum (1RM), and the number of CD68 macrophages counted on tissue sections from muscle biopsies obtained from young and elderly females, and elderly males. Spearman’s *r* and the *p*-values for the correlation are displayed for each graph. Note that if the potential outlier in the CD68 data of the elderly males is removed, the *r* value is 0.373 and the *p*-value 0.073, suggesting a trend.

Significant Spearman correlations were detected between BMI and the number of CD68+ macrophages/fiber in the control leg of the young females, and a trend was detected in the elderly females (Figure 7). This was not confirmed for the males, although removal of a potential outlier (0.11 CD68+ cells/fiber) resulted in a similar trend (*r* = 0.373, *p*-value 0.073) to that observed in the females. No significant relationship was detected between 1RM and the number of CD68+ cells/fiber in any of the groups.

## Discussion

The main findings of this study are gradually increasing numbers of macrophages and their subpopulations in response to a single bout of physiological exercise in the skeletal muscle of healthy elderly males and females, in accordance with changes in gene expression levels of inflammatory and collagen targets during the hours and days post exercise. These changes occurred from an altered local muscle inflammatory profile at baseline in elderly vs. young individuals, which together provides novel insights into the inflammatory state of aging human skeletal muscle, both at rest and when challenged with physiological muscle loading. Furthermore, we report for the first time, that the most commonly observed macrophage cell type was positive for all three markers of the macrophage markers CD68, CD11b, and CD206, suggesting that, within the physiological range of exercise-induced perturbations to healthy skeletal muscle, pure pro- or anti-inflammatory macrophages do not exist.

Studies have demonstrated that uninjured muscle in elderly individuals contains a predominance of anti-inflammatory macrophages (Cui et al., 2019; Reidy et al., 2019) which has been associated with the development of fibrosis (Mann et al., 2011) although it is possible that macrophage content increases to combat fibrosis rather than contributing to it. Indeed it has been shown that resident anti-inflammatory muscle macrophages are associated with exercise−mediated increases in skeletal muscle fiber size, suggesting a role in muscle growth (Walton et al., 2019a). Additionally a study found that anti-inflammatory macrophages in elderly are more prevalent than in young subjects prior to, and following, a muscle damaging protocol (Sorensen et al., 2019). In support of this, we detected higher levels of IL-10 and CD68 gene expression in the muscle of the control leg in the elderly compared to young females. It should also be noted that without the Bonferroni correction applied to our immunohistochemistry data, a higher content of anti-inflammatory cells (CD68+CD206+) and total CD68+ cells would have been reported in the control muscle of the elderly compared to young females, underlining that our data are not in contrast with those of Sorensen et al. (2019).

The most consistent outcome of our studies was that in the time frame of 4–7 days post exercise, we observed a clear increase in all macrophage populations in a group of 25 elderly males and 11 elderly females. This was true for both the total macrophage number, defined as CD68+ total, as well as for anti-inflammatory macrophages, defined as being either CD68+CD163+ or CD68+CD206+. The issue of molecular markers to define macrophages as pro- or anti-inflammatory is unresolved. Recently this was addressed, where it was observed that the majority of macrophages on cryo-sections of human muscle were positive for both CD11b and CD206 (Kosmac et al., 2018). Our findings are in line with this and, due to the triple labeling approached used in the current study, we add the novel insight that the most prevalent macrophage type observed was positive for CD11b, CD206, and CD68, outnumbering cells expressing CD11b or CD206 in combination with CD68. However, the findings of the majority of macrophages in human muscle being positive for both CD11b and CD206 (Kosmac et al., 2018) strongly brings into question the use of CD11b as a useful pro-inflammatory marker. Our analyses were initiated before this recent publication, where CD11b was still considered a pro-inflammatory marker, but this may be inaccurate. Similarly, our switching between CD163 and CD206 to identify anti-inflammatory macrophages in the two sets of samples was due to practical issues of needing the three primary antibodies to be from three different host species, rather than a theoretical reasoning that one was better than the other. Indeed it has been shown that the two correlate well in human muscle (Kosmac et al., 2020) so this is unlikely to make a major difference. Reconciling the various macrophage markers, and interpretation of their use, in the literature as a whole is challenging. Currently it would appear that anti-inflammatory macrophages can be identified with more confidence than pro-inflammatory macrophages, although there is clearly a large degree of overlap. It is possible that single cell, or single nuclei, RNA-sequencing of human skeletal muscle could provide greater resolution to help clarify this issue.

These findings indicate that categorizing macrophages into clearly defined pro- or anti-inflammatory subtypes may provide an inaccurate depiction of their complex functionality and how they contribute to remodeling in physiological settings. Though speculative, it is possible that the presence of pro- and anti-inflammatory markers indicates a transition from one subtype to another, or rather that most macrophages express many markers and rarely exist in pure pro- or anti-inflammatory states. It is likely that more severe models of injury induce more dramatic changes in the number of macrophages, where, given the sheer scale of tissue removal and rebuilding required, clearly distinct macrophage populations are observed (Saclier et al., 2013a). Indeed, some studies have shown that the range and duration of the presence of macrophages vary, based on the protocols used to induce muscle damage (Chazaud, 2014) raising the question regarding the role of the different macrophage subtypes present in situations of major regeneration versus tissue remodeling such as in the current study. In animals, significantly elevated concentrations of pro-inflammatory macrophages have been reported 24 h after muscle injury followed by an elevation of anti-inflammatory macrophages peaking 4–7 days post injury (Tidball and Villalta, 2010; Chazaud, 2015). Animal studies have also shown that disrupting the local transition from pro- to anti-inflammatory results in impaired or defective muscle regeneration (Saclier et al., 2013b). Another human study using eccentric muscle-damaging exercise found CD68+ cells peaking 4–7 days post exercise (Paulsen et al., 2012a) corresponding to the findings in our study. A study by Sorensen et al. (2019) investigated changes in local macrophage content following 300 lengthening contractions, a much more severe damaging model than ours, and found an increase in CD68+ cells 24H and 72H after exercise. It is likely that the reason we did not detect a significant increase at 24H post exercise is due to the lower exercise stimulus, or extent of damage, compared to that of Sorensen and colleagues. A study by Saclier et al. (2013a) in humans found that macrophage subtypes coexist in regenerative areas, where the prevalence of pro- and anti-inflammatory macrophages is determined by the phase of regeneration (early vs. late) in that specific area. In our male study we did not analyze for pro-inflammatory macrophages and therefore their presence remains unclear. Although speculative, the trend for alterations in TNF-a mRNA levels along with an immediate increase of a number of gene expression targets associated with pro-inflammatory macrophages (IL-1B, IL-1R) suggests an increased inflammatory environment prior to the increases we observed in CD163+ cells.

Regarding the resolution timeframe of tissue repair, we have previously detected elevated levels of CD68+ cells up to 30 days after using electrical stimulation to induce muscle necrosis (Mackey et al., 2011). At this time point the muscle is mostly repaired, but with clearly ongoing remodeling of the muscle connective tissue (Mackey et al., 2011, 2016) raising the possibility that macrophages exert anti-fibrotic actions, although this is speculative. Increases in COL1A1 gene expression were seen in the elderly men at 4 and 7 days post exercise. While the significance of these increased levels is difficult to interpret it indicates a heightened extracellular matrix remodeling following a heavy exercise bout. The timing of this regulation aligns with other findings from our laboratory demonstrating that ECM remodeling increases at later (>7 days) time points after exercise or injury (Mackey et al., 2016; Mackey and Kjaer, 2017; Karlsen et al., 2020). It is worth noting that 3 months after a muscle injury protocol and subsequent resistance training, COL1A1 mRNA was still upregulated in the muscle of elderly, but not in that of young, individuals (Karlsen et al., 2020) similar to the present study where greater COL1A1 mRNA levels were seen in the elderly vs. young females 5 days following the exercise bout. While the reason for this discrepancy between young and elderly is unclear it is possible that it simply represents a longer tissue healing trajectory for the older muscle.

Progressive impairment of skeletal muscle function with aging has been linked to disequilibrium between muscle damage and repair (Cui et al., 2019; Reidy et al., 2019). The findings in our study display some discrepancies between young and old subjects which may relate to this. In the elderly muscle, trends for higher content of macrophages were detected compared to the young group. While further research is necessary to fully understand the implication of these differences, it is worth noting the large spread in macrophage numbers and that we did observe a relationship between BMI and the number of macrophages (CD68+) in the control leg of the young females and a trend for a similar relationship in the elderly females (and elderly males with removal of a potential outlier). These individuals were recruited as generally healthy although there is likely to be a continuum from the higher BMI registered for some of our participants into the obese category. The high variation in macrophage content in human muscle biopsies has been reported by others, where it was found to be associated with walking performance in patients with peripheral artery disease (Kosmac et al., 2020). Similarly, the finding of an association between macrophages and muscle hypertrophy in human skeletal muscle (Walton et al., 2019b) clearly suggests a role for macrophages in the adaptive response to physiological exercise, as well as the clinical implications for macrophages in human skeletal muscle in the context of disease. Unfortunately, our study design was not suited to further explore these issues, so future work could investigate for example how the acute macrophage response to exercise is related to the hypertrophy response in the long term, and perhaps more pertinently, in the context of elderly populations.

The exercise induced increase seen for CD68+ cells and CD206+ cells in elderly females was expected and aligns with the findings observed among the male participants. Unexpectedly, no changes in macrophage counts were found in the young group, in contrast to other studies detecting increased numbers of pro-inflammatory macrophages in young individuals following a heavy resistance exercise bout (Przybyla et al., 2006; Sorensen et al., 2019). Whether our findings are related to age *per se* is difficult to determine. Indeed it is possible that muscle damage is the main driver of the macrophage response. However, we detected similar increases in circulating creatine kinase in the young and elderly females at 5 days post exercise, together with similar numbers of fibers positive for neonatal myosin at this time point (Bechshøft et al., 2019; Soendenbroe et al., 2020) suggesting that muscle damage leading to necrosis was absent. This is further supported by the complete lack of fibers containing macrophages in the present study. While these findings indicate that no major damage was induced by our protocol, we cannot rule out the possibility that a lesser response in the young females is due to the exercise stimulus being less unaccustomed due to a generally more active lifestyle. However, we did not account for this in the current study and can therefore not conclude that our findings are due to age *per se*.

Together the findings of this study provide insight into the time course of macrophage activity and associated molecular targets in the context of aging and exercise. With most of the current understanding of macrophages in skeletal muscle originating from only a handful of human studies or animal studies utilizing severe damage protocols, our findings provide a time course for macrophage infiltration in conjunction with the molecular response to a physiological bout of exercise in elderly human skeletal muscle, adding to the understanding of the interaction between macrophages, inflammation and the aging muscle. Interestingly, we report that the most prevalent macrophage phenotype present in healthy human skeletal muscle expresses multiple markers, questioning the classification into clearly defined pro- and anti-inflammatory subtypes, at least in healthy muscle where necrosis is absent. Lastly, the relationship between the muscle macrophages content in the rested state and variables such as BMI and 1RM are in line with recent work (Kosmac et al., 2020) placing muscle macrophages in a clear clinical context with regard to physical function and disease. Extricating the specific roles of macrophages in aging as such from this complex matrix requires further study.

## Data Availability Statement

The raw data supporting the conclusions of this article will be made available by the authors, without undue reservation.

## Ethics Statement

The studies involving human participants were reviewed and approved by the Committees on Health Research Ethics for the Capital Region of Denmark. The patients/participants provided their written informed consent to participate in this study.

## Author Contributions

SJ, CB, MH, PS, JA, MK, and AM contributed to the conception and design of the study. SJ, MH, and CB recruited the participants and carried out the study. SJ performed the immunohistochemical, mRNA, and statistical analyses. SJ and AM wrote the first draft of the manuscript. All the authors contributed to manuscript revision, read and approved the submitted version.

## Conflict of Interest

The authors declare that the research was conducted in the absence of any commercial or financial relationships that could be construed as a potential conflict of interest.

## Supplementary Material

FIGURE S1 Changes in muscle CD68+ cells **(top)** and CD68+CD163+ cells **(bottom)** in healthy elderly men receiving placebo (*n* = 13) or Losartan (*n* = 12) over a 7-day time course following an acute bout of heavy resistance leg extension exercise. Data were log-transformed and analyzed using a two-way repeated measures ANOVA (treatment × time) and Dunnett’s *post hoc* test to compare for an effect of time compared with PRE. Data are shown as geometric mean ± back transformed SEM. ^∗^*P* < 0.05 time compared with PRE. Tendencies are written.

FIGURE S2 Gene expression in the muscle biopsies of elderly men receiving placebo (*n* = 13) or Losartan (*n* = 12). mRNA data were normalized to RPLP0, log-transformed and are shown as geometric mean ± back transformed SEM, relative to baseline (−10 days). Data were analyzed with a two-way repeated measures ANOVA (treatment × time). ^∗^*P* < 0.05 compared with baseline. Tendencies are written.
