# Supplementary figures and images for: Macrophage Subpopulations and the Acute Inflammatory Response of Elderly Human Skeletal Muscle to Physiological Resistance Exercise
Source: Front Physiol. 2020 Jul 24;11:811. doi: 10.3389/fphys.2020.00811 (PMC7393256; doi:10.3389/fphys.2020.00811)

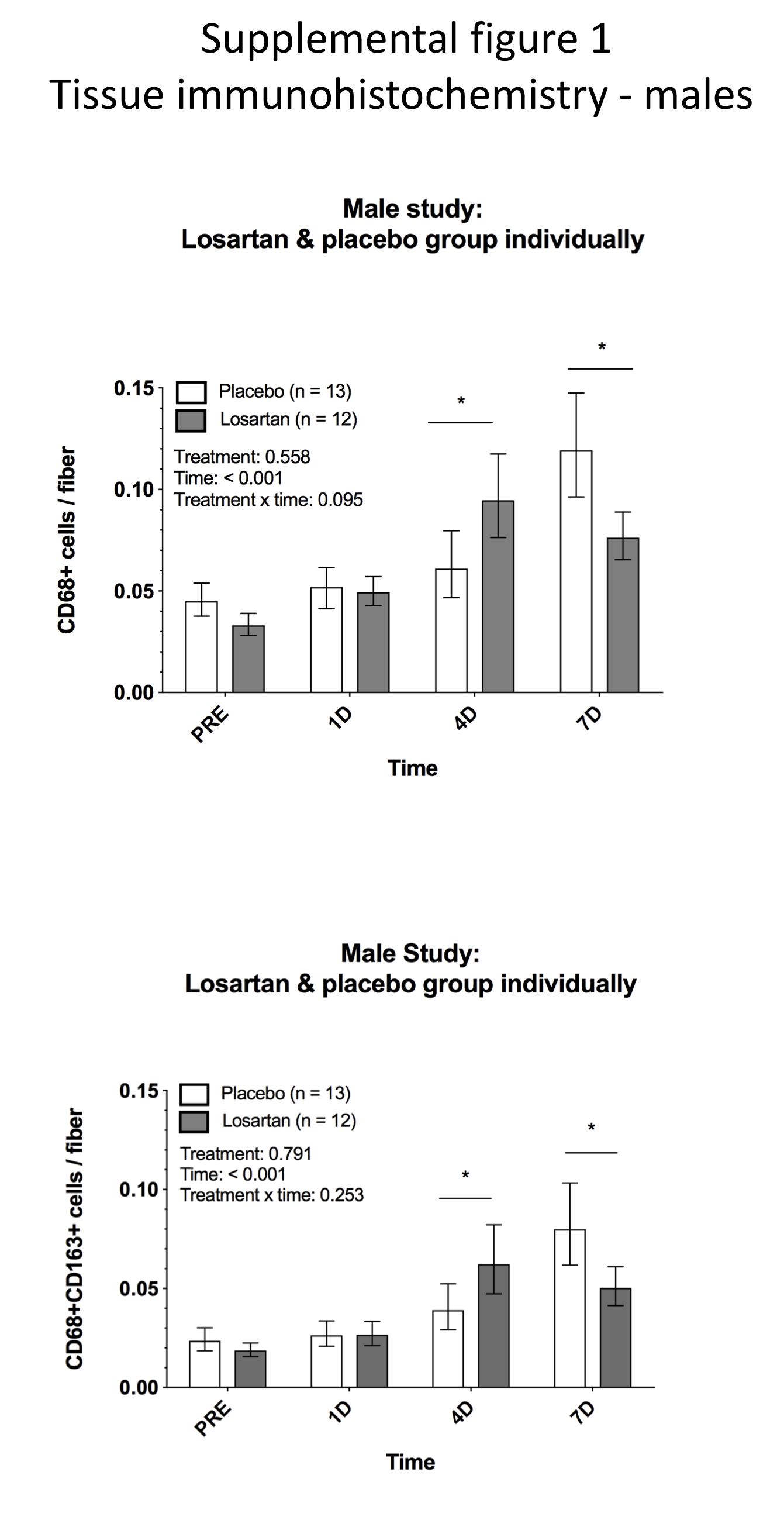

Supplement: FIGURE S1 — Changes in muscle CD68+ cells (top) and CD68+CD163+ cells (bottom) in healthy elderly men receiving placebo (n = 13) or Losartan (n = 12) over a 7-day time course following an acute bout of heavy resistance leg extension exercise. Data were log-transformed and analyzed using a two-way repeated measures ANOVA (treatment × time) and Dunnett’s post hoc test to compare for an effect of time compared with PRE. Data are shown as geometric mean ± back transformed SEM. ∗P < 0.05 time compared with PRE. Tendencies are written. [file Image_1.jpeg]

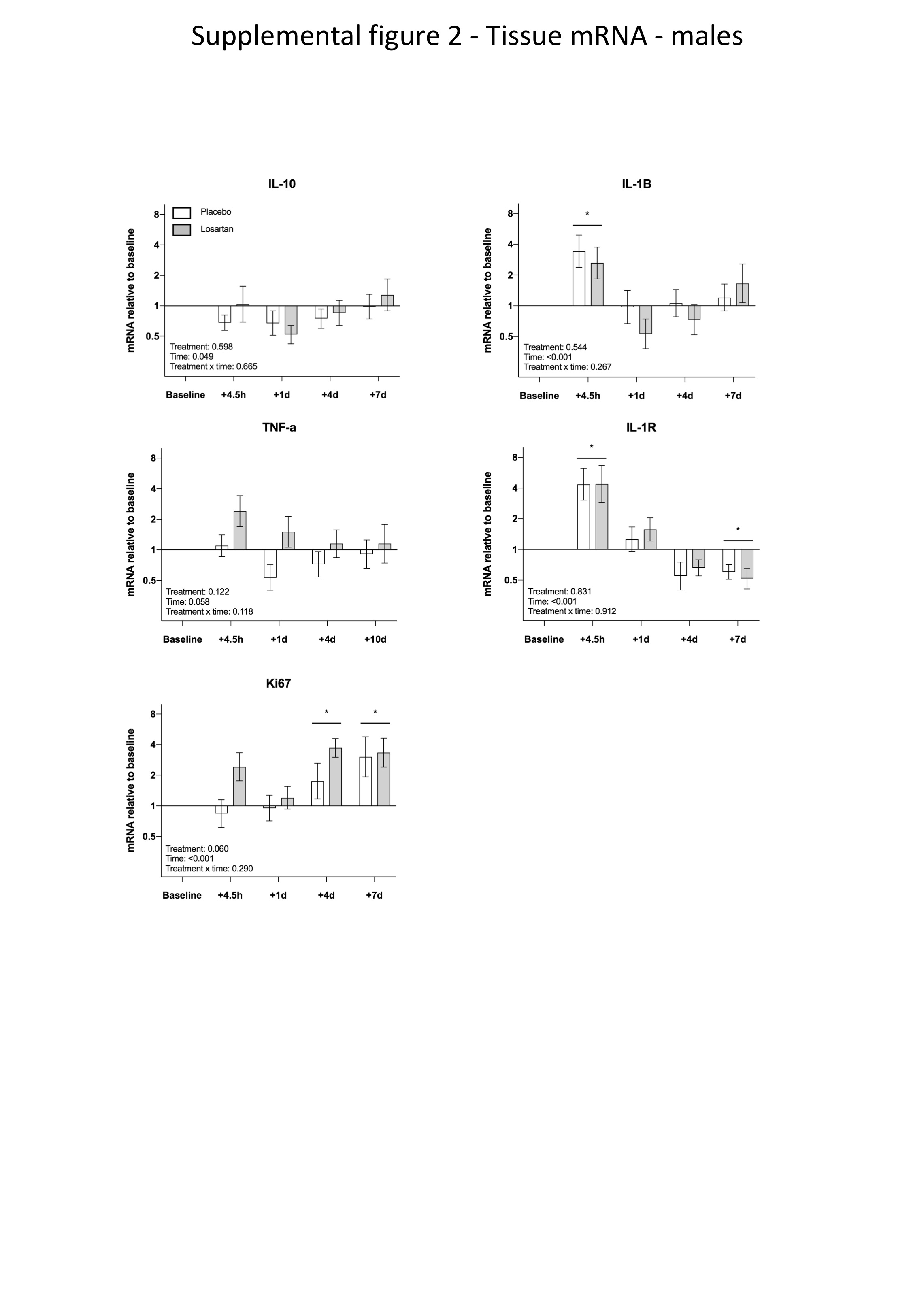

Supplement: FIGURE S2 — Gene expression in the muscle biopsies of elderly men receiving placebo (n = 13) or Losartan (n = 12). mRNA data were normalized to RPLP0, log-transformed and are shown as geometric mean ± back transformed SEM, relative to baseline (−10 days). Data were analyzed with a two-way repeated measures ANOVA (treatment × time). ∗P < 0.05 compared with baseline. Tendencies are written. [file Image_2.jpeg]
